# Sub-second Dopamine and Serotonin Signaling in Human Striatum during Perceptual Decision-Making

**DOI:** 10.1016/j.neuron.2020.09.015

**Published:** 2020-12-09

**Authors:** Dan Bang, Kenneth T. Kishida, Terry Lohrenz, Jason P. White, Adrian W. Laxton, Stephen B. Tatter, Stephen M. Fleming, P. Read Montague

**Affiliations:** 1 Wellcome Centre for Human Neuroimaging, University College London, London WC1N 3AR, UK; 2 Department of Experimental Psychology, University of Oxford, Oxford OX2 6GG, UK; 3 Department of Physiology and Pharmacology, Wake Forest School of Medicine, Winston-Salem, NC 27101, USA; 4 Department of Neurosurgery, Wake Forest School of Medicine, Winston-Salem, NC 27101, USA; 5 Fralin Biomedical Research Institute at VTC, Virginia Tech, Roanoke, VA 24016, USA; 6 Department of Experimental Psychology, University College London, London WC1H 0AP, UK; 7 Max Planck UCL Centre for Computational Psychiatry and Ageing Research, University College London, London, WC1B 5EH, UK; 8 Department of Physics, Virginia Tech, Blacksburg, VA 24061, USA

**Keywords:** dopamine, serotonin, neuromodulation, perception, decision-making, cognition, action, human, striatum, fast scan cyclic voltammetry

## Abstract

Recent animal research indicates that dopamine and serotonin, neuromodulators traditionally linked to appetitive and aversive processes, are also involved in sensory inference and decisions based on such inference. We tested this hypothesis in humans by monitoring sub-second striatal dopamine and serotonin signaling during a visual motion discrimination task that separates sensory uncertainty from decision difficulty in a factorial design. Caudate nucleus recordings (n = 4) revealed multi-scale encoding: in three participants, serotonin tracked sensory uncertainty, and, in one participant, both dopamine and serotonin tracked deviations from expected trial transitions within our factorial design. Putamen recordings (n *=* 1) supported a cognition-action separation between caudate nucleus and putamen**—**a striatal sub-division unique to primates—with both dopamine and serotonin tracking decision times. These first-of-their-kind observations in the human brain reveal a role for sub-second dopamine and serotonin signaling in non-reward-based aspects of cognition and action.

## Highlights

• Dopamine and serotonin are measured in human striatum during awake decision-making
• Serotonin tracks sensory uncertainty in caudate nucleus
• Dopamine and serotonin track sensory statistics in caudate nucleus
• Dopamine and serotonin track decision times in putamen

## Teaser

Dopamine and serotonin have traditionally been linked to reward processing. Bang, Kishida et al. show in the human brain that these neuromodulators also play a role in non-reward-based aspects of cognition and behavior, including rapid perceptual decision processes and action.

## Introduction

Neuromodulatory systems that deliver dopamine and serotonin to widespread brain structures affect basic physiological processes, including synaptic plasticity and stabilization of neural circuits (Marder, 2012). These neuromodulatory systems also participate in a variety of cognitive processes, such as motivation, mood, and learning (Cools et al., 2011; Dayan, 2012). Consistent with this broad impact on healthy function, disturbance in dopamine and serotonin signaling has been linked to diverse clinical conditions, including Parkinson’s disease (Lotharius and Brundin, 2002), anorexia nervosa (Kaye et al., 1998), obsessive compulsive disorder (Hu et al., 2006), and mood disorders such as depression (Risch et al., 2009). Yet, there are profound difficulties associated with studying neuromodulator signaling in humans (Bucher and Wightman, 2015). As a result, we have a rudimentary understanding of how neuromodulatory systems support human cognition and behavior and thereby how their dysfunction contributes to clinical conditions.

Over the last decade, this situation has begun to change, and pioneering efforts have recorded directly from human dopaminergic neurons during reward-based choice tasks (Patel et al., 2012; Zaghloul et al., 2009), although recordings from serotonergic neurons have not yet been made. Such recordings are necessary to understand how dopaminergic and serotonergic function relates to human cognition and behavior, but they will only be part of the story (Dayan, 2012). It is also necessary to measure the release-and-action of dopamine and serotonin at downstream neural targets to understand the computations that are supported by these systems and how the action of neurotransmitter agonists, antagonists, and reuptake inhibitors—drugs already in widespread use—impact human cognition and behavior (Montague and Kishida, 2018). While anatomically and chemically specific methods such as positron emission tomography (Volkow et al., 1996) and microdialysis (Meyerson et al., 1990) are available for use in humans, the recordings are on the timescale of minutes and cannot resolve the sub-second computations believed to be supported by fast neuromodulation (Dayan, 2012).

It is now possible to detect sub-second fluctuations in both dopamine and serotonin in deep structures of the human brain during conscious behavior (Kishida et al., 2011, 2016; Montague and Kishida, 2018; Moran et al., 2018). This approach involves fast scan cyclic voltammetry adapted for use in patients undergoing deep brain stimulation (DBS) surgery for disease management (e.g., Parkinson’s disease and essential tremor). To date, striatal dopamine and serotonin have been measured during a sequential investment task with variable gains and losses (Kishida et al., 2016; Moran et al., 2018). This work has produced two first-of-their-kind observations in human striatum: (1) sub-second dopamine fluctuations encode reward prediction errors, and (2) sub-second serotonin fluctuations are opponent to dopamine, showing positive transients to negative reward prediction errors and negative transients to positive reward prediction errors. However, the task is a low-dimensional probe of the dopaminergic and serotonergic systems—involving a single valence axis ranging from punishment to reward—and it is unclear how these systems function in more complex settings, such as perceptual decision-making, where variables relating to sensation, action, and learning are simultaneously at play.

Pharmacological manipulations in humans (Crockett et al., 2012; Guitart-Masip et al., 2012) and recordings or perturbations of neuronal activity in animals (Fonseca et al., 2015; da Silva et al., 2018) already indicate that the dopaminergic and serotonergic systems support not only valence processing but also behavioral control—with dopamine invigorating and serotonin inhibiting responses. Further, recent animal research suggests that these systems support an even broader set of computations. For example, dopamine may track an animal’s strength of belief about sensory states (Lak et al., 2017) and surprise about non-reward-related features of sensory stimuli (Takahashi et al., 2017), whereas serotonin may track an animal’s uncertainty about task rules (Iigaya et al., 2018) and promote behavioral persistence in the face of short-term negative outcomes (Lottem et al., 2018). These developments promise to help advance our understanding of how the dopaminergic and serotonergic systems support healthy function in humans, but they also raise the issue of how to generalize insights from model organisms to humans (Montague and Kishida, 2018). There is an urgent need for similarly rigorous work in the conscious human brain where experimental paradigms can be even further refined to probe granular aspects of cognition and behavior.

**Figure 1:**
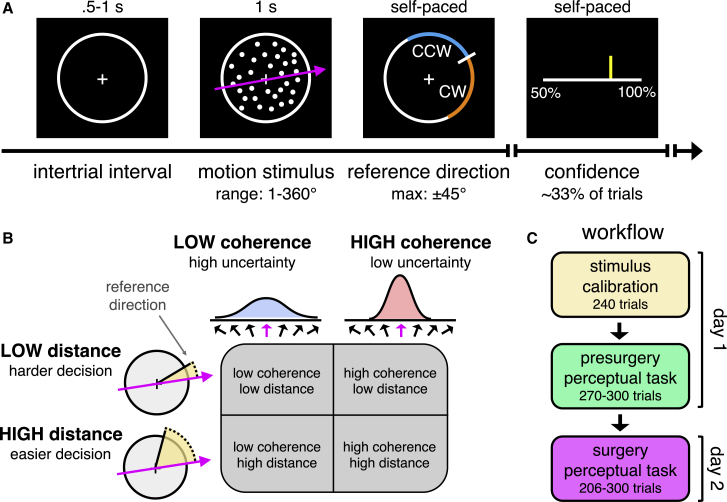
Experimental Framework (A) Continuous direction random dot motion task with variable reference. Participants had to judge whether the net direction of dot motion (sampled from the range 1–360°) was counterclockwise (CCW) or clockwise (CW) to a reference direction that appeared after stimulus offset (maximum absolute angular distance from motion direction: 45°). On around a third of trials, participants were required to estimate their confidence in the perceptual decision on a discrete visual scale indicating probability correct (50% to 100% in steps of 10%). Participant 5 viewed the motion stimulus for 0.8 s on day 2 due to a different configuration of the display monitor in the operating room. (B) Factorial design. We varied the fraction of coherently moving dots (sensory uncertainty) and the absolute angular distance between the motion direction and the reference direction (decision difficulty) in a two-by-two design. (C) Workflow. On day 1, we calibrated stimulus parameters to achieve target levels of performance and trained participants on the task. On day 2, we measured dopamine and serotonin fluctuations while participants performed the task during neurosurgery.

We deployed a visual perceptual decision task while recording sub-second changes in dopamine and serotonin delivery to human striatum (caudate nucleus and putamen). The task, adapted from the standard random dot motion paradigm (Newsome et al., 1989), requires participants to judge the average direction of dot motion relative to a reference direction, which only appears at the offset of the motion stimulus (Figure 1A). In addition to this temporal dissociation of sensory inference and decision formation, sensory uncertainty can be separated from decision difficulty by independently varying the fraction of coherently moving dots and the distance between the average motion direction and the reference direction (Figure 1B) (Bang and Fleming, 2018). For example, a participant may have low uncertainty about the average direction of dot motion (high coherence) but find it hard to judge their motion percept against the reference direction (low distance). The task thus allowed us to study the joint contribution of dopamine and serotonin at both the input and the output level of decision-making and within highly integrative neural structures. Critically, the striatum is believed to support perceptual decision-making (Cox and Witten, 2019; Hanks and Summerfield, 2017), and the random dot motion task has been shown to activate the striatum in both non-human primates and humans (Bang and Fleming, 2018; Ding and Gold, 2010; Doi et al., 2020). To anticipate our results, we show that dopamine and serotonin track within-trial variables relating to uncertainty and action as well as cross-trial variables relating to task statistics.

## Results

Participants (Parkinson’s disease, n = 2; essential tremor, n = 3) performed the task on two separate days (Figure 1C). On day 1, during a presurgical visit, we first calibrated the stimulus parameters and then trained participants on the task. The purpose of the stimulus calibration was to normalize perceptual experience across participants and to control for any potential influence of disease state on perceptual decision-making (Huang et al., 2015; Matthews et al., 2020). On day 2, as part of the neurosurgical procedure for implantation of a DBS electrode, participants performed the task in the operating room while we simultaneously measured dopamine and serotonin levels in sub-structures of the striatum (caudate nucleus, n = 4; putamen, n = 1) with sub-second temporal resolution (10 Hz). The fast scan cyclic voltammetry protocol used to obtain these measurements is described in STAR Methods (see also Figure S4 for illustration of electrochemical approach and Figure S5 for evaluation of sensitivity and specificity to dopamine and serotonin against a background of varying pH).

### Behavioral Separation of Sensory Inference from Decision Formation

**Figure 2:**
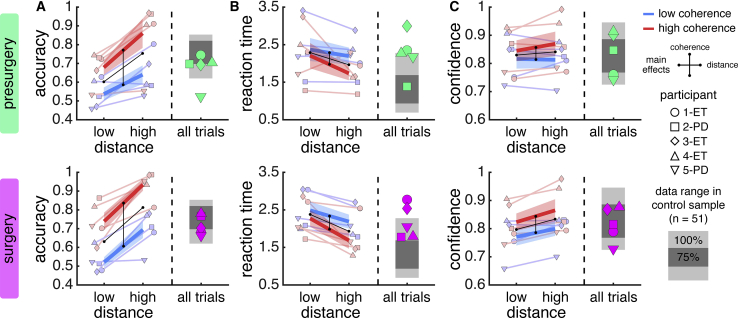
Behavioral Performance Behavioral data shown separately for presurgery (top, green) and surgery (bottom, pink). (A) Choice accuracy. (B) Choice reaction time as measured from onset of reference direction. (C) Confidence estimates as elicited on around a third of trials. In (A)–(C), blue lines denote low coherence and red lines denote high coherence. Black lines indicate main effects (vertical: low versus high coherence; horizontal: low versus high distance). Shaded gray bands denote the ranges of behavioral data observed in a non-patient sample. Data are represented as group mean ± SEM. Symbols indicate participant number and disease state (PD: Parkinson’s disease. ET: essential tremor). See Table S1 for regression statistics and analysis split by session.

Our task aimed to separate sensory inference from decision formation by (1) varying the uncertainty of a motion percept (coherence) independently of the difficulty of a judgement based on this motion percept (distance) and (2) segregating these processes in time (reference direction appears after the offset of the motion stimulus). As intended, participants’ behavioral responses were affected by both coherence and distance. In particular, participants made more accurate choices when coherence was high and when distance was high (Figure 2A, hierarchical logistic regression; coherence: t_2727_ = 4.76, p < 0.001, distance: *t*_2727_ = 3.46, p = 0.001, interaction: t_2727_ = 2.65, p = 0.008). These effects were mirrored in choice reaction time, with participants making faster choices when coherence was high and when distance was high (Figure 2B; hierarchical linear regression; coherence: t_2727_ = −3.58, p < 0.001, distance: t_2727_ = −3.57, p < 0.001, interaction: t_2727_ = −1.30, p = 0.195). In support of our task rationale, this variation in task performance was reflected in subjective experience—as measured by responses on the visual confidence scale (Figure 1A) —with participants reporting higher confidence when coherence was high and when distance was high (Figure 2C, hierarchical linear regression; coherence: t_908_ = 2.30, p = 0.022, distance: *t*_908_ = 2.44, p = 0.015, interaction: t_908_ = 0.81, p = 0.418). Overall, participants’ behavioral responses were well matched before and during surgery (compare rows in Figure 2) —an important observation given the special setting of the operating room. Further, as expected given the stimulus calibration, participants’ behavioral responses fell within the range of responses displayed by healthy controls (n = 51; gray bands in Figure 2).

### Caudate Nucleus and Putamen

In primates, the dorsal striatum is divided into the caudate nucleus and putamen by a white-matter structure known as the internal capsule. The functional significance of this division is debated (Haber, 2016), but one prominent hypothesis, supported by circuit tracing in non-human primates (Alexander et al., 1986; Middleton and Strick, 2000) and MRI-based resting-state connectivity and diffusion tractography in humans (Leh et al., 2007; Di Martino et al., 2008), is that the division, at least in part, reflects a cognition-action axis. In particular, it has been hypothesized that the caudate nucleus and putamen are part of distinct striato-thalamo-cortical loops: (1) a *cognition* loop passing largely through the caudate nucleus, receiving inputs from association areas and ultimately returning outputs to prefrontal cortex, and (2) an *action* loop passing largely through the putamen, receiving inputs from sensorimotor areas and ultimately returning outputs to premotor areas (Alexander et al., 1986; Middleton and Strick, 2000). Our results were consistent with a functional separation between the caudate nucleus and putamen, and we have organized the paper accordingly.

We measured dopamine and serotonin signaling in the caudate nucleus of four participants and in the putamen of one participant (see Figure S1 for electrode coordinates). First, in caudate nucleus recordings from participants 1–3, we show that serotonin tracks sensory uncertainty within a trial as probed by our manipulation of coherence. Further, in caudate nucleus recordings from participant 4, we show that both dopamine and serotonin track deviations from expected trial transitions between the four conditions of our factorial design—the reason why we analyzed their data separately from the other caudate nucleus participants. Second, in putamen recordings from participant 5, we show that dopamine and serotonin track choice submission—as revealed by ramping profiles leading up to a choice that were stable across variation in choice accuracy and choice reaction time.

### Caudate Nucleus: Serotonin Signaling Tracks Sensory Uncertainty

As supported by the behavioral results, our task separates the uncertainty about a sensory stimulus (coherence) from the difficulty associated with a subsequent decision about this stimulus (distance). Recent research in model organisms indicates that the dopaminergic and serotonergic systems carry information about these variables. There is evidence that the activity of dopaminergic neurons tracks an animal’s *certainty* (i.e., increase in firing rate indicates higher certainty) about sensory states when faced with ambiguous sensory information (Lak et al., 2017, Lak et al., 2020; Starkweather et al., 2017). There is also evidence that the activity of serotonergic neurons tracks an animal’s *uncertainty* (i.e., increase in firing rate indicates higher uncertainty) (Iigaya et al., 2018; Lottem et al., 2018; Matias et al., 2017). However, this hypothesis about serotonin signaling has only been tested in the context of reward-based probabilistic learning across many trials and not perceptual decision-making where uncertainty unfolds on a moment-by-moment basis within single trials. More broadly, this work on dopaminergic and serotonergic signaling has recorded or perturbed neuronal activity, and it remains to be seen whether neuromodulator release at target sites displays similar computational motifs.

**Figure 3:**
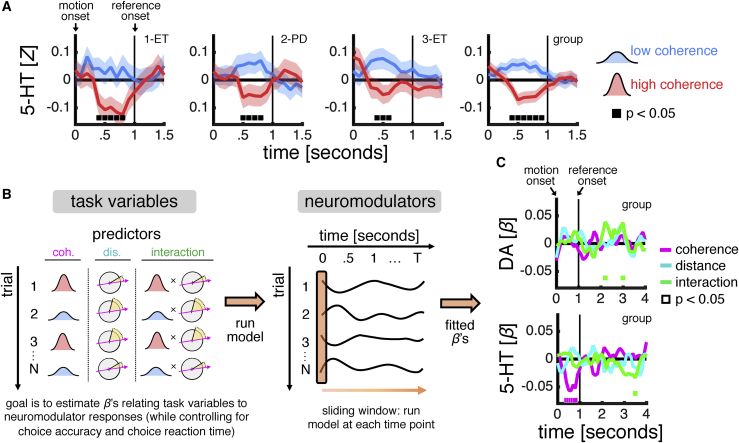
Caudate Nucleus: Serotonin Signaling Tracks Sensory Uncertainty (A) Serotonin time series from caudate nucleus grouped by level of coherence in participants 1–3. Marker indicates that time series for low coherence (blue) and high coherence (red) are statistically different (p < 0.05, independent-samples t test). Data are represented as mean ± SEM. See Figure S2A for dopamine time series and Figure S2B for an analysis of dopamine and serotonin responses to motion coherence as a function of the laterality of the motion direction with respect to the hemisphere in which the recording electrode is located. (B) Schematic of sliding-window regression approach used to quantify relationship between task variables and neuromodulatory responses. We deployed a multiple linear regression across all trials where we predicted neuromodulatory responses at each time point (sliding window indicated by orange band) using coherence (pink) and distance (cyan) as well as their interaction (green). We included choice accuracy and choice reaction time as nuisance variables (not shown). All predictors were *Z* scored. The estimated regression coefficients (*β*’s) quantifies the encoding of task variables in neuromodulatory responses. (C) Dopamine and serotonin encoding profiles from caudate nucleus in participants 1–3. Marker indicates that a coefficient is statistically different from zero (p < 0.05) as estimated by the regression approach described in (B). Group-level analysis was conducted by combining data across participants and including a random intercept for each participant. See Figure S2C for individual participants. In (A) and (C), time series were locked to the onset of the motion stimulus, spanning a period from 1 s before stimulus onset to 5 s after stimulus onset, *Z* scored separately for each trial, and smoothed using a running average (.5 s). Top right-hand corner indicates participant number and disease state (PD: Parkinson’s disease; ET: essential tremor). The fourth caudate nucleus participant was not included in this analysis because their neuromodulatory responses—as explained in the main text—were qualitatively different. DA: dopamine. 5-HT: serotonin.

In our study, we separated sensory uncertainty from decision difficulty by (1) varying the fraction of coherently moving dots independently from the distance between the average motion direction and the reference direction and (2) segregating these variables in time such that sensory inference—as probed by changes in coherence—can be analyzed separately from decision formation—as probed by changes in distance. Following this rationale, we first assessed the impact of sensory uncertainty on neuromodulator signaling by grouping dopamine and serotonin responses to the motion stimulus according to the level of coherence. In caudate nucleus recordings from participants 1–3, this analysis revealed that serotonin tracked sensory uncertainty: shortly after stimulus onset, there was a transient increase in serotonin when coherence was low and a transient decrease in serotonin when coherence was high, both at the group level and in individual participants (compare blue and red lines in Figure 3A). By contrast, there was no consistent relationship between dopamine and sensory uncertainty across participants 1–3 (Figure S2A). This result provides the first human evidence that serotonin signaling tracks uncertainty and extends this computational motif to a short-lived sensory stimulus upon which a perceptual judgement is based.

Next, expanding our time window of interest beyond stimulus presentation, we extended our analysis to include decision difficulty. To separate the contributions of our task variables to neuromodulatory responses using a single analysis framework, we performed a sliding-window regression (Figure 3B). At each time point, we predicted neuromodulatory responses across trials using the levels of coherence and distance as well as their interaction while controlling for choice accuracy and choice reaction time. This procedure returns encoding profiles that quantify the relationship between specific task variables and neuromodulatory responses across time. We note that, given the sequential nature of our task and the randomization of task conditions, an encoding of distance, or an encoding of the interaction between coherence and distance, should only be seen after the onset of the reference direction.

At the group level, in caudate nucleus recordings from participants 1–3, the sliding-window regression (Figure 3C) indicated that serotonin, in addition to an expected main effect of coherence during stimulus presentation (pink), tracked an interaction between coherence and distance (green) around 2.5 s after the reference direction was revealed. The fitted coefficients indicated that the interaction effect was driven by a transient *decrease* in serotonin for the easiest trial type defined by high coherence and high distance. Dopamine, which did not show a main effect of coherence during stimulus presentation (pink), tracked an interaction between coherence and distance (green) around 1 s, and again around 2 s, after the onset of the reference direction. In contrast to the encoding profile of serotonin, the fitted coefficients indicated that the interaction effect was driven by a transient *increase* in dopamine for the easiest trial type. We caution that the interaction effects, unlike the main effect of coherence on serotonin, were not sustained in time and varied between participants (Figure S2C). Taken together, these results support a hypothesis that serotonin signaling tracks sensory uncertainty and provide preliminary human evidence that dopamine and serotonin signaling tracks information relating to the overall probability that a perceptual judgement is correct.

### Caudate Nucleus: Dopamine and Serotonin Signaling Tracks Experienced Trial Type Transitions

**Figure 4:**
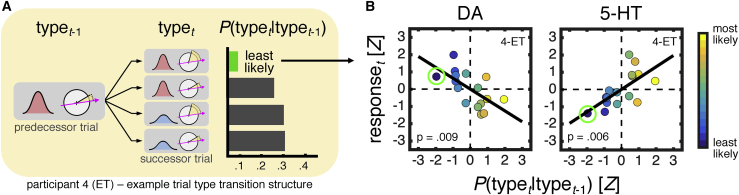
Caudate Nucleus: Dopamine and Serotonin Signaling Tracks Experienced Trial Type Transitions (A) Illustration of experienced task statistics for participant 4. Horizontal bars show the probability of encountering each trial type on trial *t* after having encountered the trial type defined by high coherence and low distance on trial *t*-1. The trial type transition probabilities, *P*(type_*t*_|type_*t*-1_), were computed as the normalized counts of the number of times each trial type succeeded a given trial type. The expected value of *P*(type_*t*_|type_*t*-1_) under randomization is 0.25. The least likely trial type transition is indicated by green color in (A) and (B). (B) Correlation between neuromodulatory responses and trial type transition probabilities in participant 4. To compute the neuromodulatory responses, we first averaged time points across a window from 0 s to 1.5 s (the period during which a trial type is revealed) within each trial and then averaged across all trials for each trial type transition. Time series were locked to the onset of the motion stimulus, spanning a period from 1 s before stimulus onset to 5 s after stimulus onset, and *Z* scored separately for each trial. For comparison between dopamine and serotonin, data points are colored according to the associated trial type transition probability. Lines are best-fitting lines from a linear regression. Top right-hand corner indicates participant number and disease state (PD: Parkinson’s disease; ET: essential tremor). DA: dopamine. 5-HT: serotonin.

In participants 1–3, the encoding profiles indicate that the neuromodulatory responses are *evoked* by the presentation of the task variables. However, in the fourth caudate nucleus participant, both dopamine and serotonin appeared to carry information about the task variables at the start of a trial (see sliding-window regression in Figure S2C) —a result that led us to separate their data from the other caudate nucleus participants. We suspected that both neuromodulators were sensitive to experienced dependencies between trials and pursued an analysis strategy based on this hypothesis. Our task turns the standard random dot motion task into a discrete two-by-two design —crossing coherence (low versus high) and distance (low versus high). There are thus four trial types, each of which may be followed by another of the same four trial types, yielding a total of sixteen trial type transitions. Trial types were sampled randomly, but the experienced probability of transitioning between two trial types may nevertheless differ from the probability expected under randomization (i.e., 0.25). Figure 4A provides an example of experienced task statistics for participant 4—here showing the probability of a trial type on the current trial (trial *t*) given that the trial type on the previous trial (trial *t*-1) was defined by high coherence and low distance. In this example, one of the trial type transitions is less likely than the others (compare green bar to rest) and would therefore be more surprising from a statistical learning perspective (see Figure S3 for full transition matrix).

To assess the contribution of such experienced task statistics to dopamine and serotonin signaling, we performed a linear regression in which we predicted neuromodulatory responses to trial type transitions based on the associated trial type transition probabilities. To obtain a trial-level estimate of the neuromodulatory response for each trial type transition, we averaged across all time points from the onset of the motion stimulus to half a second after the onset of the reference direction—the time period during which the current trial type is revealed. This analysis showed that both dopamine and serotonin tracked the probability of the current trial type conditional on the previous trial type—with lower dopamine and higher serotonin for less likely trial type transitions (Figure 4B; dopamine, t_14_ = −3.03, p = 0.009; serotonin, *t*_14_ = 3.24, p = 0.006). These relationships remained after we controlled for the marginal probability of a trial type and the choice accuracy associated with a trial type transition (not shown; dopamine, t_12_ = −2.92, p = 0.013; serotonin, t_12_ = 2.89, p = 0.014) —supporting an interpretation that the neuromodulatory responses reflected experienced task statistics and not sequential effects on task performance. Taken together, these results provide preliminary evidence for an impact of statistical structure on dopamine and serotonin signaling (see Discussion for possible explanations such as a direct role in statistical learning). We note that the qualitatively distinct neuromodulatory responses in the fourth caudate nucleus participants compared to the other caudate nucleus participants are unlikely to be due to differences in task performance (similar to rest as shown in Figure 2), disease state (participant 2 is also an essential tremor patient), and/or the electrode location within the caudate nucleus (similar to rest as shown in Figure 1S).

### Putamen: Dopamine and Serotonin Signaling Tracks Choice Submission

**Figure 5:**
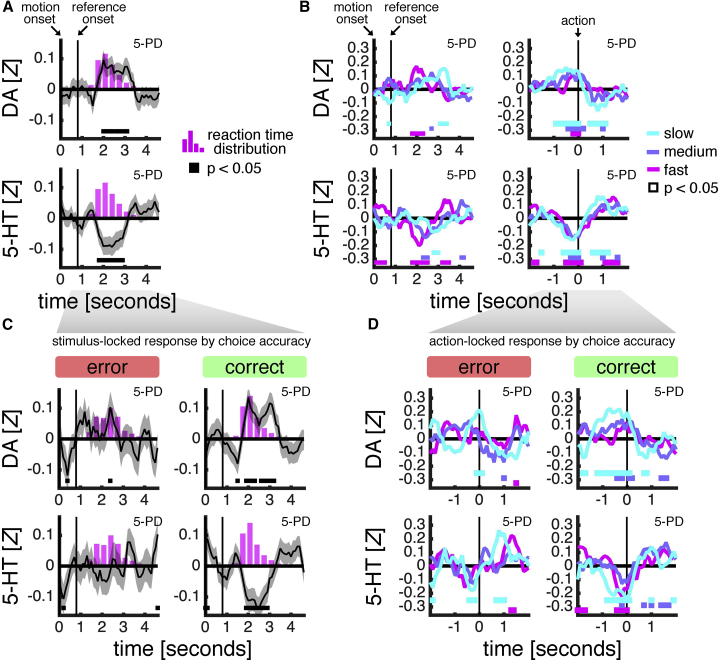
Putamen: Dopamine and Serotonin Signaling Tracks Choice Submission (A) Dopamine and serotonin time series from putamen locked to the onset of the motion stimulus and overlaid onto distribution over choice reaction time (pink histogram) in participant 5. See Figure S2D for participants 1–4. (B) Dopamine and serotonin time series from putamen locked to stimulus onset (left) or choice submission (right) and grouped by terciles over choice reaction time in participant 5. (C) Same as in (A) but separated by choice accuracy. (D) Same as in right-hand side of (B) but separated by choice accuracy. In (A)–(D), time series were *Z* scored separately for each trial (stimulus-locked: period spanned from 1 s before stimulus onset to 5 s after stimulus onset; choice-locked: period spanned from 4 s before choice submission to 4 s after choice submission) and smoothed using a running average (.5 s). Data are represented as mean ± SEM in (A) and (C) and as mean in (B) and (D). Marker indicates that a time point is statistically different from zero (p < 0.05, one-sample t test). Top right-hand corner indicates participant number and disease state (PD: Parkinson’s disease; ET: essential tremor). DA: dopamine. 5-HT: serotonin.

We next turned to the fifth participant—the only participant where we recorded from the putamen and not the caudate nucleus. Anatomical and functional assays indicate that a division of primate dorsal striatum into the caudate nucleus and putamen reflects, at least in part, a cognition-action axis (Alexander et al., 1986; Middleton and Strick, 2000). In line with this functional separation, in putamen recordings from participant 5, but not in caudate nucleus recordings from participants 1–4, dopamine and serotonin responses were aligned to the moment at which a choice was submitted. In particular, the aggregate neuromodulatory responses locked to the onset of the motion stimulus tracked the distribution over choice reaction time—with dopamine ramping up and serotonin ramping down (compare black traces to pink histogram in Figure 5A) —a pattern not observed in caudate nucleus recordings from participants 1–4 (Figure S2D). The close coupling between changes in neuromodulator delivery to the putamen and choice submission indicates that both dopamine and serotonin play a role in triggering action but in an opponent manner.

If changes in dopamine and serotonin delivery to the putamen trigger action, then we would expect these changes to happen earlier for faster choices. Indeed, when grouping neuromodulatory responses by terciles over choice reaction time, the earliest peaks were observed for the fastest set of choices: serotonin displayed temporally distinct downward peaks, whereas dopamine, moving in a direction opposite to serotonin, displayed more sustained responses for each choice reaction time tercile (left-hand panels in Figure 5B). One pertinent question is whether the neuromodulators reach a fixed level prior to choice regardless of the time taken to make a choice—akin to a fixed bound in sequential sampling models (Kiani and Shadlen, 2009) —or whether the level required to trigger a choice decays across time—akin to a collapsing bound (Drugowitsch et al., 2012). When locking neuromodulatory responses to choice submission, both dopamine and serotonin reached a fixed level prior to choice: serotonin displayed rapid downward transients before choice submission—transients that were aligned across all choice reaction time terciles—whereas dopamine showed a more languid upward transient for the slowest set of choices (right-hand panels in Figure 5B).

Finally, we asked whether these choice-related responses varied with choice accuracy. When locking neuromodulatory responses to the onset of the motion stimulus, there was a noticeable difference between correct and error trials, with dopamine and serotonin tracking the distribution over choice reaction time on correct trials only (Figure 5C). However, error trials are associated with more variable choice reaction times (compare pink histograms in Figure 5C) —partly due to the fact that incorrect choices reflect a broader mixture of events including distraction and motor errors—variability that may obscure a choice-related signal in neuromodulatory responses locked to the onset of the motion stimulus. Indeed, when locking responses to the moment at which a choice was made, dopamine and serotonin displayed ramping profiles on error as well as correct trials, although this pattern was more distinct on correct trials (Figure 5D). The relative accuracy-invariance of the response patterns support an interpretation that changes in dopamine and serotonin delivery to the putamen are involved in triggering choice rather than tracking some other feature of the choice process.

## Discussion

The dopaminergic and serotonergic systems are believed to be essential for basic neural and cognitive processes (Dayan, 2012; Marder, 2012). However, our understanding of these systems has been impeded by a lack of chemically specific methods for studying neuromodulation in humans at fast timescales. Using fast scan cyclic voltammetry adapted for use in neurosurgical patients (Kishida et al., 2011, 2016; Montague and Kishida, 2018; Moran et al., 2018), we measured for the first time sub-second changes in dopamine and serotonin delivery to human striatum during a visual perceptual decision task—a commonly used laboratory system for studying cognition in humans and non-human primates (Shadlen and Kiani, 2013). Our results reveal that sub-second dopamine and serotonin signaling in human striatum participates in real-time inference about the external world—beyond their often-reported roles in appetitive and aversive processes.

By augmenting the standard random dot motion paradigm, we were able to separate the uncertainty about a sensory stimulus from the difficulty associated with a decision about this stimulus. In caudate nucleus recordings, we found that serotonin tracked sensory uncertainty—as revealed by a transient increase to low-coherence stimuli and a transient decrease to high-coherence stimuli. This function for sub-second serotonin signaling has never before been reported in humans. In model organisms, serotonin has been linked to uncertainty, but in the context of reward-based probabilistic learning (Iigaya et al., 2018; Lottem et al., 2018). However, in contrast to this work where uncertainty is defined for a variable that operates across trials, we show that serotonin tracks uncertainty about a variable—a short-lived sensory stimulus—that unfolds within single trials. Taken together, these findings indicate that serotonin tracks uncertainty at multiple levels of abstraction.

Several animal studies have already investigated dopamine in the context of perceptual decision-making (Lak et al., 2017, Lak et al., 2020; Nomoto et al., 2010). One of these studies, using the standard random dot motion paradigm, found that the firing rate of dopaminergic neurons in the monkey midbrain scaled with the level of coherence leading up to a choice, which, if correct, resulted in the delivery of a juice reward (Nomoto et al., 2010). Re-analysis of these data using a reinforcement learning model whose value predictions incorporated sensory uncertainty indicated that the dopaminergic neurons encoded the probability of a reward given sensory evidence and choice (Lak et al., 2017). Our study complements this work. First, our study was conducted in humans. Second, we recorded dopamine fluctuations at target sites as opposed to spike modulation at parent cell bodies—addressing the issue of how computational variables hypothesized to be encoded by midbrain dopaminergic neurons are transformed at the downstream site of release where local modulation of synaptic terminals is likely to occur (Montague et al., 2004a, 2004b).

In caudate nucleus recordings, we found no consistent relationship between dopamine and sensory uncertainty across participants. One potential reason is that our task—unlike the standard random dot motion paradigm—separates sensory uncertainty from decision difficulty. Indeed, by applying a sliding-window regression that included both of these task variables as well as their interaction, we found preliminary evidence that dopamine tracked—in a manner opponent to serotonin—information relating to the overall probability that a perceptual decision is correct—as indicated by an interaction effect between sensory uncertainty and decision difficulty. This result, albeit not as robust as the effect of sensory uncertainty on serotonin, fits with the computational characterization of monkey midbrain dopaminergic neurons on the standard random dot motion paradigm (Lak et al., 2017). Our task, however, did not involve trial-by-trial feedback (or reward), suggesting that dopamine may also code for the *intrinsic* value of making a correct choice. We highlight that we observed more individual variation in dopamine than serotonin responses (Figure S2). Interestingly, simultaneous recording of dopaminergic neurons in the rat midbrain has shown that distinct clusters of neurons encode distinct aspects of behavior and cognition (Engelhard et al., 2019) —a spatial and functional specialization that may give rise to subtle differences in dopamine release across recording sites in the striatum and thereby complicate group-level analysis of dopamine signaling across participants.

In the early reward prediction error models of dopamine, value predictions are computed on the basis of a perfect representation of the state of the world (Montague et al., 1993, Montague et al., 1996). In recent years, this model has been extended to incorporate state uncertainty by assuming that value computation operates on an inferred distribution of hidden states given ambiguous cues (Gershman and Uchida, 2019) —an assumption that is needed to account for the response profile of dopaminergic neurons under direct manipulations of state uncertainty (Lak et al., 2017; Starkweather et al., 2017). In parallel, it has been proposed that dopamine encodes a generalized prediction error that trains a statistical model of the world (Gardner et al., 2018) —a proposal that is supported by preliminary evidence that dopamine tracks changes in value-neutral features of rewards (Takahashi et al., 2017) and is causal to the learning of stimulus-stimulus associations (Sharpe et al., 2017, 2020).

While not directly probed by our task design, the data from one caudate nucleus participant support a hypothesis that dopamine is involved in, or at least is modulated by, statistical learning. In particular, we found that dopamine responses scaled with the experienced probability of encountering a state on the current trial conditional on the state encountered on the previous trial (here, state is one of the four conditions in our factorial design). Intriguingly, serotonin responses scaled with the experienced state transition probabilities in a manner that mirrored dopamine—potentially extending the opponency between dopamine and serotonin to statistical learning. Our task does not allow us to isolate the computational mechanism driving these relationships—the neuromodulatory responses may reflect prediction errors on state transitions or some cognitive construct (e.g., preparation for particular trial types) that is modulated by task statistics. Future studies that directly manipulate task statistics is needed to address the relationship of dopamine and serotonin signaling to statistical learning.

It is now generally agreed that dopamine and serotonin code both for valence (reward or punishment) and action (invigoration or inhibition) (Boureau and Dayan, 2011; Crockett et al., 2012; Fonseca et al., 2015; Guitart-Masip et al., 2012; da Silva et al., 2018). However, to date, there has been no human work on the link between phasic neuromodulation and action and, despite their hypothesized opponency, no concurrent measurements of dopamine and serotonin in relation to action. In the context of a visual perceptual decision task, we found, in putamen recordings, strong evidence in support of opposing roles of dopamine and serotonin in relation to action. Specifically, the submission of a choice was preceded by a transient increase in dopamine, consistent with dopamine promoting action (“pressing the accelerator”), and a transient decrease in serotonin, consistent with inhibitory effects of serotonin on action being turned off (“releasing the brake”). These response profiles were only seen in the putamen and not in the caudate nucleus—a division that may reflect a cognition-action axis within dorsal striatum (Alexander et al., 1986; Middleton and Strick, 2000). We acknowledge that our putamen recordings were obtained in a single participant—due to the putamen being located along a less common trajectory in DBS surgery—and that further recordings from the putamen are needed in order to draw definitive conclusions about functional stratification within dorsal striatum.

Our study was conducted in patients diagnosed with Parkinson’s disease or essential tremor, but there are several reasons why our results are likely to generalize to the healthy brain. Unlike Parkinson’s disease (Poewe et al., 2017; Wilson et al., 2019), essential tremor involves small or no disturbances in the dopaminergic or serotonergic systems (Benito-León and Louis, 2006). The fact that our key result—that serotonin tracks sensory uncertainty—was found in not only patients with essential tremor but also a patient with Parkinson’s disease supports a hypothesis that neuromodulator measurements in either disease state provide a window onto the healthy brain. Further, the degeneration of midbrain dopaminergic neurons in Parkinson’s disease is believed to affect tonic dopamine levels (Poewe et al., 2017) and may thus have had a diminished impact on phasic fluctuations as examined here. However, phasic disturbances in Parkinson’s disease remain an open question—new insights into disease etiology may be provided by using fast scan cyclic voltammetry during DBS surgery for other conditions such as obsessive compulsive disorder and treatment-resistant depression (Holtzheimer and Mayberg, 2011) and then comparing neuromodulatory responses between disease states. Finally, we note that the patients received medications for disease management (see Table S2). However, the medications that target neuromodulation are administered to alter tonic levels and are not widely believed to alter phasic fluctuations as studied here. In further support of the generality of our findings, our key result—that serotonin tracks sensory uncertainty—was found in patients with different medication profiles.

One inherent constraint on all fast scan cyclic voltammetry experiments is the requisite use of *in vitro* measurements to make predictions about *in vivo* data. Our approach—utilizing large *in vitro* datasets to fit cross-validated penalised regression models for *in vivo* prediction—begins to address rigor and reproducibility on this issue. Under our approach, positive identification of an analyte is objectively determined and does not require visual confirmation by an experienced investigator. We have shown that this approach outperforms traditional methods for dopamine prediction (Kishida et al., 2016) and that it can identify and separate dopamine and serotonin under standard voltammetry protocols for data acquisition (Moran et al., 2018). One limitation of any quantitative approach to fast scan cyclic voltammetry is that the prediction models can only explicitly account for analytes and conditions used to train the models; in other words, the prediction models are biased by the training data. However, our approach retains this information. In contrast, traditional methods (Keithley and Wightman, 2011) that require visual inspection of the data (e.g., background-subtracted voltammograms) introduce untraceable bias inherent to any subjective assessment. Sources of variance explicitly accounted for in our training data are electrode drift, subtle variations in electrode construction, changes in pH, and a wide range of biologically plausible dopamine and serotonin concentrations. We demonstrate that these training data result in high specificity and sensitivity for dopamine and serotonin measurements made on naive probes withheld from training (Figure S5). That being shown, we cannot fully rule out that the *in vivo* dopamine and serotonin measurements and resulting predictions are contaminated by other neurochemicals present in brain tissue such as uric acid, adenosine, ascorbate, 3,4-dihydroxyphenylacetic acid (DOPAC), 5-hydroxyindoleacetic acid (5-HIAA), 3-mothoxytyramine (3-MT), and homovanillic acid (HVA). There is currently no evidence to suggest that these neurochemicals play a role in perceptual decision-making on the timescales that we report, but future work may nevertheless reveal a role for any of them. Critically, our approach allows for re-interrogation of existing datasets with targeted training data. In order to facilitate such use of the current data, and data analysis using other approaches, we have made the raw data from each patient freely available (see Data and Code Availability).

In summary, our results support a view that sub-second dopamine and serotonin signaling participates in real-time inference about the external world (Dayan, 2012; Gershman and Uchida, 2019). Further, we observed in some, but not all cases, opponent dynamics between dopamine and serotonin. An opponent coding scheme may at times be redundant, but it also provides computational benefits, such as fault tolerance and high-dimensional representations of the external world (e.g., many objects contain both positive and negative features but these are not always equally relevant and should be dissociated) (Montague et al., 2016). However, given the diversity of cell and receptor types (Andrade and Haj-Dahmane, 2013; Berke, 2018; Civelli et al., 1993; Gaspar and Lillesaar, 2012; Julius, 1991; Ranade and Mainen, 2009; Roeper, 2013), and the ability of dopamine and serotonin to cross-load onto each other’s terminals (Carta et al., 2007; Gantz et al., 2015; Zhou et al., 2005), opponent dynamics may be subtle and vary within the brain. We acknowledge that other neuromodulators such as norepinephrine are involved in perceptual decision-making at fast timescales (Aston-Jones and Cohen, 2005) but, excitingly, proof-of-concept work shows that it is possible to detect norepinephrine and distinguish it from dopamine with a quantitative approach to fast scan cyclic voltammetry (Montague and Kishida, 2018). Further, proof-of-concept work shows that neuromodulator measurements can be obtained from the depth electrodes that are routinely used in epilepsy monitoring—opening up for investigation in a variety of neural structures (Montague and Kishida, 2018). In this connection, one target for future research is the human adaption of silicon-based microelectrode arrays (Taylor et al., 2019) that allow for multi-site coverage within a given structure. Overall, our study opens the door to a deeper understanding of neuromodulatory systems that have remained poorly understood due to a lack of chemically specific methods for fast neuromodulator measurements in humans.

## STAR★Methods

### Key Resources Table

**Table:** 

REAGENT or RESOURCE	SOURCE	IDENTIFIER
**Chemicals, Peptides, and Recombinant Proteins**		

Dopamine: DA	Sigma-Aldrich	H8502; CAS: 62-31-7
Serotonin: 5-HT	Sigma-Aldrich	H9523; CAS: 153-98-0
Reagent for PBS: NaCl	Sigma-Aldrich	S7653-1K; CAS: 7647-14-5
Reagent for PBS: KCl	Sigma-Aldrich	P9333-1K; CAS: 447-40-7
Reagent for PBS: Na2HPO	Sigma-Aldrich	S7907-1K; CAS: 7558-79-4
Reagent for PBS: KH2PO4	Sigma-Aldrich	P5655-1K; CAS: 7778-77-0

**Deposited Data**		

Raw current time series	This paper	https://osf.io/qyv9b/
Dopamine and serotonin predictions	This paper	https://github.com/danbang/article-DA-5HT-perceptual-decision

**Software and Algorithms**		

MATLAB	MathWorks	MATLAB R2015B
Cogent 2000	UCL	http://www.vislab.ucl.ac.uk/cogent_2000.php
Glmnet	Qian et al., 2013	http://web.stanford.edu/∼hastie/glmnet_matlab/
pCLAMP	Molecular Devices	pCLAMP 10 Axon Instruments
Custom code for main analyses	This paper	https://github.com/danbang/article-DA-5HT-perceptual-decision

**Other**		

Carbon-fiber microelectrodes	Kishida et al., 2016	In-house custom-made electrodes
Amplifier	Molecular Devices	Multiclamp 700B Axon Instruments
Head stage	Molecular Devices	CV-7B-EC Axon Instruments
A/D converter	Molecular Devices	Digidata 1440A Axon Instruments
Signal generator	Tektronix	AFG320
Isolation transformer	Tripp Lite	IS500HG Isolation Transformer

### Resource Availability

#### Lead Contact

Further information and requests for resources should be directed to and will be fulfilled by the Lead Contact, Dan Bang (danbang.db@gmail.com).

#### Materials Availability

This study did not generate new unique reagents.

#### Data and Code Availability

Data and code supporting main results are available at GitHub (https://github.com/danbang/article-DA-5HT-perceptual-decision). Raw current time series from each patient are available at the Open Science Framework (https://osf.io/qyv9b/).

### Experimental Model and Subject Details

#### Patients

Five patients (1 female, age range: 67-81, mean age ± SD: 73.40 ± 5.55 years) participated in the study. Patients were diagnosed with Parkinson’s disease (n = 2) or essential tremor (n = 3) and deemed good candidates for DBS treatment. Once they had agreed to the clinical procedure, they were assessed for the research study and given the option to participate. Before obtaining informed written consent, the research protocol and how it would alter the clinical procedure were explained – specifically, that the procedure would involve an additional research-exclusive probe (the carbon-fiber microelectrode) and that extra time (maximum 30 min) would be needed to complete the research protocol. This information was provided both verbally and in a written document. Once informed written consent had been obtained, patients proceeded with the research study, involving first a behavioral training session and then a surgical test session. During surgery, patients sat in a semi-upright position and viewed a monitor at a distance of approximately 100 cm. Patients used a gamepad to submit responses. Dopamine replacement medications used to treat disease symptoms were withheld from the day before surgery as per standard of care (see Table S2 for medications). No adverse or unanticipated events occurred during or as a result of the described procedures. The study was approved by IRB committees at Wake Forest University Health Sciences (IRB00017138) and Virginia Tech (IRB 11-078).

#### Controls

A cohort of fifty-one adults (25 females, age range: 19-64 years, mean age ± SD: 35.49 ± 12.81 years) were recruited as healthy controls (i.e., no reported history of psychiatric or neurological disorder). They performed a behavioral training session and subsequently an fMRI test session – here, we only report data from the behavioral training session as this session was fully matched between patients and controls. All controls provided informed written consent. The study was approved by the IRB committee at Virginia Tech (IRB 11-078).

### Method Details

#### Visual perceptual decision task

##### Task description and factorial design

Participants performed a continuous direction variable reference random dot motion task as shown in Figure 1A. Each trial began with the presentation of a fixation cross at the center of a circular aperture. After a uniformly sampled delay (.5-1 s), participants viewed a field of moving dots (1 s; 0.8 s for participant 5 as the display monitor in the operating room unintendedly had a higher refresh rate). On each update of the display, a fraction of dots moved coherently in a specified direction, sampled anew on each trial from the range 1-360°, whereas the remainder moved randomly. Once the stimulus terminated, participants were presented with a reference direction which transected the aperture. Participants were required to press one of two buttons to indicate whether the average direction of dot motion was counterclockwise (CCW) or clockwise (CCW) to the reference (angle originating in center). The arrangement of the average motion direction and the reference direction (i.e., whether the correct decision was CCW or CW) was sampled randomly on each trial. As a visual aid, the response buttons and the associated arcs of the aperture were colored orange and blue (color assignment counterbalanced across participants). Once a choice had been made, the color of the central cross (orange or blue) indicated the decision (.25 s). On approximately a third of trials, participants were then asked to indicate their confidence in the perceptual decision on a discrete visual scale indicating probability correct (50% to 100% in steps of 10%). A confidence marker started randomly in one of the six locations along the scale and was controlled by button press. Once a response had been submitted, the marker turned gray (.5 s), before the next trial started. On the rest of the trials, participants proceeded directly to the next trial after having made a decision.

Using a factorial design as illustrated in Figure 1B, we independently varied the fraction of coherently moving dots (low or high coherence) and the absolute angular distance between the average motion direction and the reference direction (low or high distance). This design separates sensory uncertainty (coherence) from decision difficulty (distance). For example, a participant may have low uncertainty about the average direction of dot motion (high coherence) but find it hard to judge their motion percept against the reference direction (low distance). Conversely, a participant may have high uncertainty about the average direction of dot motion (low coherence) but find it easy to judge their motion percept against the reference direction (high distance). The levels of coherence (low or high) and distance (low or high) were sampled randomly on each trial.

##### Stimulus specification

The motion stimulus was made up of three sets of dots (each dot was 0.12 degrees in diameter) shown in consecutive frames inside the circular aperture (8 degrees in diameter) centered on the fixation cross (0.2 degrees in diameter). Each set of dots was shown for one frame (about 16 ms) and then replotted again three frames later (about 50 ms) – some dots were displaced in the specified motion direction at a speed of 2 degrees s^-1^ while the rest of the dots were displaced at random locations within the aperture. We refer to the percentage of dots displayed in the specified motion direction as coherence, C. The dot density was fixed at 16 dots degrees^-2^ s^-1^. These details were subtly different for participant 5 as the display monitor in the operating room unintendedly had a higher refresh rate (72 Hz instead of 60 Hz). To help subjects maintain fixation, a circular region (0.7 degrees in diameter) at the center of the aperture was kept free of dots. The motion direction was sampled uniformly from the range 1-360 degrees. The direction of the reference (0.8 degrees in length and 0.08 degrees in width) was within ±45 degrees of the motion direction. We refer to the absolute angular difference between the specified motion direction and the reference direction as distance, D. A pair of coherences, **C**, and a pair of distances, **D**, were calibrated for each participant.

##### Stimulus calibration

The aim of the stimulus calibration was to identify a pair of coherences associated with different levels of sensory uncertainty and a pair of distances associated with different levels of decision difficulty. In order to minimize the time needed for stimulus calibration during the presurgical visit, we used an interleaved procedure, calibrating coherence (C) and distance (D) in alternate blocks of trials. We used a “two-down-one-up” procedure whereby the parameter being calibrated was decreased after two correct decisions and increased after one incorrect decision (C: ±0.01; D: ±1). In coherence blocks, we calibrated a medium coherence (C_M_) at a medium distance (D_M_) and used this value to specify low coherence (C_L_ = C_M_ × 0.5) and high coherence (C_H_ = C_M_ × 2). In distance blocks, we separately calibrated low distance (D_L_) and high distance (D_H_) at high coherence and low coherence, respectively. Under the ”two- down-one-up” procedure, this separation should yield around 71% choice accuracy in the C_L_D_H_ and C_H_D_L_ conditions. Medium coherence was initialised at 0.3 and otherwise defined as the mean of low and high coherence (C_M_ = C_L_ × 0.5 + C_H_ × 0.5). Medium distance was initialised at 20 and otherwise defined as the mean of low and high distance (D_M_ = D_L_ × 0.5 + D_H_ × 0.5). This procedure returned a pair of coherences, **C**: {C_L_, C_H_}, and a pair of distances, **D**: {D_L_, D_H_}, that were individually customised to each participant. Because the surgical session did not allow additional time for stimulus calibration, the same stimulus parameters were used in the presurgical and the surgical sessions.

#### Workflow

Participants performed a behavioral training session and a surgical test session on separate days. The behavioral training session had three phases. In phase 1, participants received on-screen instructions and practised the task (40 trials). In this phase only, participants received trial-by-trial feedback about choice accuracy. Further, coherence and distance were fixed at high levels (coherence = 0.5; distance = 30), with the aim to familiarise participants with making direction judgements in continuous space. In phase 2, participants performed the task (240 trials) while we calibrated a pair of coherences and a pair of distances so as to achieve target levels of task performance. In phase 3, participants performed the task (270-300 trials) including intermittent confidence reports. The surgical test session had two phases. In phase 1, during an electrochemical conditioning protocol, participants received on-screen instructions and viewed example motion stimuli. In phase 2, participants performed the task (206-300 trials) including intermittent confidence reports.

#### Electrochemical approach

Here we first provide a general description of our approach, before detailing its implementation in the current study.

##### General description

Our approach builds on fast scan cyclic voltammetry (FSCV) as applied to model organisms and model systems (e.g., slices or cultures) over the last three decades (Bucher and Wightman, 2015; Rodeberg et al., 2017). Our carbon-fiber microelectrodes are made in the same way as those used in rodents (Clark et al., 2010) and we have only modified their dimensions for use in the human brain (Kishida et al., 2016). We have validated in the rat brain – by stimulating dopamine axons into the striatum – that our electrodes have similar electrochemical properties to rodent electrodes (Kishida et al., 2011). Our data acquisition protocol is identical to that used in rodent studies with regard to the time course of the voltage sweeps and the recording of the induced current time series during those sweeps (Phillips et al., 2003). The only change is the statistical method used to estimate the concentration of analytes of interest from the current time series measured by the electrode in the patient brain. As we explain below, this approach is optimized for human electrochemistry where an electrode cannot be used for analyte calibration prior to or after the surgical procedure due to contamination issues.

FSCV involves the delivery of a rapid change in electrical potential to an electrode and measurement of the induced electrochemical reactions as changes in current at the electrode – with the guiding idea being that the current response carries information about both the identity and the concentration of analytes in the surrounding neural tissue. The goal of analysis of FSCV data is therefore to develop a statistical model that uses the current response in the best possible way to separate and estimate analytes of interest (here dopamine and serotonin against a background of varying pH). The standard procedure – which we also use here – is to train the statistical model on FSCV data collected in an *in vitro* environment where the presence and concentration of analytes of interest can be controlled and then apply this model to FSCV data collected *in vivo* for analyte inference. As explained above, human studies require that different electrodes are used to collect *in vitro* and *in vivo* data.

Traditionally, the statistical model involves a decomposition of the *in vitro* training data into principal components that are then used for *in vivo* analyte inference within a regression framework (Heien et al., 2004). In broad terms, this approach treats analyte inference as a problem of signal reconstruction: the concentration of an analyte of interest is estimating by mapping an *in vivo* current response onto those collected in the controlled *in vitro* environment and then using the best match to label the *in vivo* current response. We instead treat analyte inference as a problem of signal prediction – with the statistical model optimized to generate accurate predictions about out-of-training data (e.g., a dopamine concentration measured at another time point or on another electrode). This step is achieved by training an elastic net regression model – a standard machine learning method (Zou and Hastie, 2005) – on non-decomposed *in vitro* data such that every single time point within a current time series contributes to signal prediction (see illustration in Figure S4). In support of this approach, visualization of model parameters shows that analyte information is distributed throughout a current time series and not only at the oxidation or reduction peaks typically revealed by principal components analysis (Figure S4E). To facilitate out-of-training signal prediction – the only option in human electrochemistry where analyte inference must be performed for an out-of-training electrode – we train the statistical model with cross-validation and use *in vitro* data that are orders of magnitude larger than those typically used (in terms of density of concentrations sampled, number of replicate measurements per concentration and number of electrodes used).

There are statistical advantages to this approach to analyte inference. First, cross-validated training mitigates against any bias in the assembly of the training data, it prevents against overfitting to the training data, and it allows for objective assessment of the contribution of model parameters to signal prediction (Figure S4E). Second, reframing analyte inference as a problem of signal prediction means that the statistical model can be directly evaluated using *in vitro* data that were withheld from training (Figure S5). Third, an objective classification approach sidesteps the need for experimenter judgement (e.g., the cut-off for the number of principal components based on their reconstructed variance) and visual assessment of current responses (e.g., visualization of background-subtracted voltammograms).

We have previously validated the approach in several ways. First, we have shown that the approach returns more reliable dopamine estimates than principal component regression (Kishida et al., 2016). Second, we have shown (and do so again in Figure S5) that our approach can separate dopamine and serotonin (Moran et al., 2018). Third, we have shown (and do so again in Figure S5) that our approach does not confuse changes in pH for changes in dopamine or serotonin (Kishida et al., 2016; Moran et al., 2018). Fourth, we have shown that our approach can separate dopamine and serotonin from other analytes such as 5-HIAAA – a serotonin metabolite – and norepinephrine (Montague and Kishida, 2018). More broadly, an objective classification approach opens the door for quantitative assessment of FSCV for analytes that are difficult (if not impossible) to detect by visual assessment of current responses (e.g., norepinephrine) and re-interrogation of *in vivo* data (such as the data collected here or the rich model organism literature) with targeted *in vitro* training data.

We acknowledge that “interferents” – that is, other neurochemicals present in brain tissue such as pH, uric acid, adenosine, ascorbate, 3,4-dihydroxyphenylacetic acid (DOPAC), 5-hydroxyindoleacetic acid (5-HIAA), 3-mothoxytyramine (3-MT), and homovanillic acid (HVA) – are a challenge for any electrochemical technique and an active area of research. Our *in vitro* training data was designed to facilitate separate estimation of changes in dopamine and serotonin against a background of varying pH. There are several reasons for this focus. First, in contrast to dopamine and serotonin, there is no a prior rationale in the literature to suggest that the interferents listed above play a role in rapid sensory inference, decision formation, and/or statistical learning. Second, compared to pH, dopamine, and serotonin, these interferents are formed by biological processes that operate on slower timescales (e.g., minutes). In this way, while they may pose a challenge in a static environment, these interferents are unlikely to have confounded the estimation of changes in dopamine or serotonin at sub-second timescales. We highlight that the current time series from each patient are freely available and any interested researcher will therefore be able to test hypotheses about potential interferents with targeted *in vitro* training data.

##### FSCV carbon-fiber microelectrodes

As part of the surgical procedure to implant a DBS electrode, we recorded dopamine and serotonin release using FSCV on a custom-made carbon-fiber microelectrode. The carbon-fiber microelectrode was inserted into the dorsal striatum along a guide cannula which was positioned in accordance with DBS planning. The carbon-fiber microelectrode was constructed to have the same dimensions as the tungsten microelectrode used for functional DBS mapping. The recording site depended on the DBS target (see Figure S1 for electrode coordinates). Probe construction and the mobile electrochemical recording station are described in detail in previous work (Kishida et al., 2011, Kishida et al., 2016).

##### FSCV protocol

Our FSCV protocol follows earlier work in rodents (Clark et al., 2010; Phillips et al., 2003) and humans (Kishida et al., 2011, 2016; Moran et al., 2018). Our measurement waveform was a standard triangular voltage ramp (ramp up from −0.6 V to +1.4 V at 400 V/s, ramp down from +1.4 V to −0.6 V at −400 V/s). While patients were prepared to perform the task in the surgical suite, we ran a conditioning protocol consisting of a 60 Hz application of the measurement waveform (hold at −0.6 V for 6.67 ms, ramp up to +1.4 V at 400V/s, ramp down to −0.6 V at −400 V/s, and repeat) for around 10 min in order to allow equilibration of the recording surface. Then, during the task, a 10 Hz application of the measurement waveform was applied for the entire duration of the experiment (hold at −0.6 V for 90 ms, ramp up to +1.4 V at 400 V/s, ramp down to −0.6 V at −400 V/s, and repeat) with a base 100 KHz sampling rate. Examples of (A) the application of the measurement waveform, (B) the resulting voltammogram, and (C) its derivative, which we use for analyte inference, are shown in Figure S4.

##### Dopamine-serotonin prediction model

We estimated dopamine and serotonin concentrations every 100 ms (10 Hz) from the *in vivo* FSCV data using multivariate regression models that were trained and cross-validated on *in vitro* FSCV data containing labeled concentrations of dopamine and serotonin in the presence of varying pH. Training and cross-validation was performing using the elastic net algorithm – an automatic shrinkage and regularization approach to fitting regression models (Zou and Hastie, 2005) – as implemented in the *glmnet* package for MATLAB (Qian et al., 2013).

##### *In vitro* training data

The *in vitro* training data was based on a population of 20 probes (carbon-fiber microelectrodes). Each probe contributed three datasets: one in which DA is varied from 0 to 4500 nM, one in which 5-HT is varied from 0 to 4500 nM, and one in which pH is varied from 6.9 to 7.8. We created a pooled training dataset by randomly sampling an equal number of samples from each probe (2500 DA, 2500 5-HT, and 500 pH) – where a sample is a voltammogram recorded at 100 KHz during the 10 ms triangular voltage waveform portion of the measurement waveform (1000 time points). To avoid equilibration and flow artifacts, samples were only taken from the third quarter of the timeline for the recording of a given analyte solution. The pooled dataset covered the ranges for DA and 5-HT with up to 50 nM resolution and the range for pH with up to 0.1 pH resolution.

##### *In vitro* model training

We divided the concentration ranges for DA and 5-HT into five non-overlapping ranges that spanned 900 nM each – thereby forming a 5×5 grid, $r_{i,j}$, of concentration ranges for model training, where i indicates the range of DA and j indicates the range of 5-HT (Table S3). For each $r_{i,j}$, we then assembled a dataset of voltammograms, $x_{i,j,n}$, and concentration labels, $y_{i,j,p,n}$, by including the appropriate ranges of DA and 5-HT data and a full range of pH data (i.e., the same pH data was used for each $r_{i,j}$), where n indicates an individual sample and p indicates the analyte.

Using these training datasets, we optimized linear multivariate regression models for each $r_{i,j}$ using the elastic net algorithm (Qian et al., 2013; Zou and Hastie, 2005). More specifically, we predicted each concentration label, $y_{i,j,p,n}$, using the derivative of each voltammogram,$\;x_{i,j,n}$. Thus, for each $r_{i,j}$, we had dependent variables, which were contained in an N×P (samples by analytes) matrix of concentration values, and predictor variables, which were contained in an N×Q matrix (where Q=1000−1 given a sampling rate of 100 KHz over the 10 ms triangular voltage waveform portion of the measurement waveform). The elastic net algorithm for regression models fits a set of parameters, β, to the time points within the differentiated voltammogram by minimizing the residual sum of squares with an additional penalty term, $P_{\alpha }\left(\beta \right)$. The elastic net penalty, $P_{\alpha }\left(\beta \right)=\left(1-\alpha \right)1/2\beta_{\ell_{2}}^{2}+\;\alpha \beta_{\ell_{1}}$, is a mixture of the ridge regression penalty ($\ell_{2}-norm:1/2\beta_{\ell_{2}}^{2}$) (Hoerl and Kennard, 1970) and the lasso penalty ($\ell_{1}-norm:\beta_{\ell_{1}}$) (Tibshirani, 1996) parameterized by α, which takes values between 0 and 1. For each $r_{i,j}$, we fitted 11 linear multivariate regression models – each with α set to a value of $\alpha_{k}$ on the 0-1 range in steps of 0.1 – using 10-fold cross-validation. This procedure yielded a 5 (DA training range) x 5 (5-HT training range) x 11 (α) cube, $F_{i,j,k}$:

$$\text{glmnet}\;\text{command}:F_{i,j,k}=\mathrm{cvglmnet}\left(\mathrm{diff}\left(x_{i,j}\right),y_{i,j}{,}^{\prime }{\mathrm{mgaussian}}^{\prime },\alpha_{k},\ldots \right)$$

We selected the best-fitting model $M_{i,j}$ for each $r_{i,j}$ by finding the $F_{i,j,k}$ with the lowest mean cross-validated error.

We highlight that we do not fit a single model to the full concentration ranges but instead fit separate models to sub-divisions of the full range. Model training at one range is therefore unaffected by the existence of other ranges within the training data. In this way, we do not commit to any notion of “biological range” and avoid biasing our results toward any particular range – a methodologically cautious approach given that “biological range” may not generalize from model organisms to the human brain and may vary between structures within the human brain.

##### *In vivo* prediction generation

For each *in vivo* dataset X containing N voltammograms, we used each $M_{i,j}$ to create a 5×5 grid of concentration predictions $Y_{i,j}$:

$$\text{glmnet}\;\text{command}:Y_{i,j}=\text{cvglmnetPredict}\left(M_{i,j},\;\text{diff}\left(X\right),{\;}^{\prime }\text{lambda}\_{\text{min}}^{\prime }\right)$$

Here, each $Y_{i,j}$ contained N predictions for DA, 5-HT, and pH. We then calculated the error $e_{i,j}$ between $Y_{i,j}$ and the mean value of the training concentration range $r_{i,j}$ for DA and 5-HT (pH was ignored):

$${\text{e}}_{i\;j,DA}=\left\{ \begin{array}{cc} \sum_{n=1}^{N}{\left(Y_{i\;j,DA,n}-\bar{r_{i\;j,DA}}\right)}^{2} & if\;\text{mode}\;\left(\text{sign}\left(Y_{i\;j,DA}\right)\right)\neq -1\\ \infty & otherwise \end{array}\right.$$

$${\text{e}}_{i\;j,5HT}=\left\{ \begin{array}{cc} \sum_{n=1}^{N}{\left(Y_{i\;j,5HT,n}-\bar{r_{i\;j,5HT}}\right)}^{2} & if\;\text{mode}\;\left(\text{sign}\left(Y_{i\;j,5HT}\right)\right)\neq -1\\ \infty & otherwise \end{array}\right.$$

$${\text{e}}_{i\;j}={\text{e}}_{i\;j,DA}+{\text{e}}_{i\;j,5HT}$$

The $Y_{i,j}$ with the minimum $e_{i,j}$ was then chosen as the predictions for dataset X. This step was done as a given model generally produces more accurate predictions when the concentration ranges are matched between training and test data. Finally, we used the center of the 10 ms triangular voltage waveform portion of the measurement waveform for voltammogram $X_{n}$ as the timestamp for prediction $Y_{n}$ on the experimental timeline.

##### Model evaluation

We evaluated the specificity and sensitivity of the prediction model using *in vitro* datasets from six naive probes that were withheld from model training (Figure S5).

The first three probes (probes A-C) contributed datasets that were collected in the same way as those used for model training. We divided the DA and 5-HT datasets from the probes into three parts – each spanning a range of 1500 nM – as a given *in vivo* dataset is unlikely to span the full range. We created predictions for each dataset and evaluated model performance by plotting predicted versus known concentrations. As shown in Figure S5A, the prediction model performed well in this out-of-sample test scenario. The relationship between predicted and known concentrations is overall linear, but the exact scaling of this relationship does not impact our results – our claims are based on relative changes at short timescales around events of interest.

The remaining three probes (probes D-F) contributed datasets that were collected in solutions with a mixture of dopamine and serotonin against a stable background of pH – with each probe exposed to a unique range of dopamine and serotonin. As shown in Figure S5B, the prediction model performed well in this mixed out-of-sample test scenario.

##### Prediction normalization

We present *in vivo* dopamine and serotonin concentration estimates from the prediction model as *Z* scores. There are several reasons why normalization is desirable. First, our research question concerns transient changes that occur within a trial – such as the response to the presentation of the motion stimulus or the submission of a choice about this stimulus. In order to detect these transient changes, we subtract the average response within a trial – the background against which the transient changes occur. This step also facilitates comparison between trials as the background response itself may vary across trials. For example, neuromodulatory systems are critical for general brain function and may fluctuate – at slower timescales – with physiological or cognitive states that are not relevant to our task. Furthermore, the current response of the electrode – and thus the concentration estimates – may drift across time – like any other neural signal. Second, we want to compare transient changes not only across trials but also across participants. In order to facilitate such comparison, we divide the mean-subtracted responses by the variability of responses within a trial. Potential sources of variance across participants include baseline dopamine and serotonin levels and the physical distance between the electrode and sites of neuromodulator release (see Table S4 for range of changes within each participant). As shown in Figure S4F, normalization does not affect the shape of the neuromodulator time series but brings them into a common frame of reference for data analysis.

#### Data analysis

##### Trial exclusion

We excluded trials from behavioral and neuromodulator analysis in which the choice reaction time was 3 SD below or above the average choice reaction time within a session. This procedure resulted in the exclusion of approximately 2% of trials per subject. In addition, we excluded the first and the last trial of each session from behavioral and neuromodulator analysis.

##### Behavioral analysis

Statistical analysis of behavior was based on trial-by-trial data pooled across the behavioral and the surgical sessions. We used hierarchical mixed-effects regression as implemented by MATLAB’s *fitglme* function to predict choice accuracy (logistic), choice reaction time (linear), and confidence reports (linear). We first log-transformed choice reaction time and then *Z* scored all variables except for choice accuracy separately for each session. We modeled participant-level slopes and intercepts, and report statistics at the group level. The distribution of residuals was assumed to be normal, but this was not formally tested. Regression coefficients and analysis split by session are reported in Table S1.

##### Neuromodulator analysis

Statistical analysis of neuromodulatory response was based on single-trial snippets constructed from the time series generated by the dopamine-serotonin prediction model. We constructed stimulus-locked time series (lasting from 1 s before stimulus onset to 5 s after stimulus onset) and choice-locked time series (lasting from 4 s before choice to 4 s after choice). We *Z* scored the time series for each trial using the mean and standard deviation across a trial and smoothed the normalized time series using a 0.5 s sliding window (i.e., the sliding-window estimate for time point *t* is the average over time points *t*-4 to *t*). Statistical testing was performed by (1) comparing concentrations between two conditions at time point *t* using an independent-samples t test (e.g., Figure 3A), (2) comparing concentrations at time point *t* to zero using a one-sample t test (e.g., Figure 5) or (3) applying multiple linear regression at each time point *t* using *Z* scored predictors of interest (e.g., Figure 3C). Group-level analysis was conducted by pooling data across participants and including a random intercept for each participant. Statistical testing was not corrected for multiple comparisons (time points) in this first human study probing neuromodulator fluctuations during a visual perceptual decision task. We do not yet have substantial prior models of what the dopaminergic and serotonergic systems encode during such tasks – hence the novelty of the current steps to understand this new domain of perceptual decision-making – and thus we had no principled way to decide on natural signal classes or null conditions.

## Supplemental Information

Document S1. Figures S1–S5 and Tables S1–S4

Document S2. Article plus Supplemental Information
