# Supplementary material for: Sub-second Dopamine and Serotonin Signaling in Human Striatum during Perceptual Decision-Making
Source: Neuron. 2020 Dec 9;108(5):999–1010.e6. doi: 10.1016/j.neuron.2020.09.015 (PMC7736619; doi:10.1016/j.neuron.2020.09.015)
Supplement: Document S1. Figures S1–S5 and Tables S1–S4 [file mmc1.pdf]

Neuron, Volume *108*

Supplemental Information

Sub-second Dopamine and Serotonin Signaling in Human Striatum during Perceptual Decision-Making

Dan Bang, Kenneth T. Kishida, Terry Lohrenz, Jason P. White, Adrian W. Laxton, Stephen B. Tatter, Stephen M. Fleming, and P. Read Montague

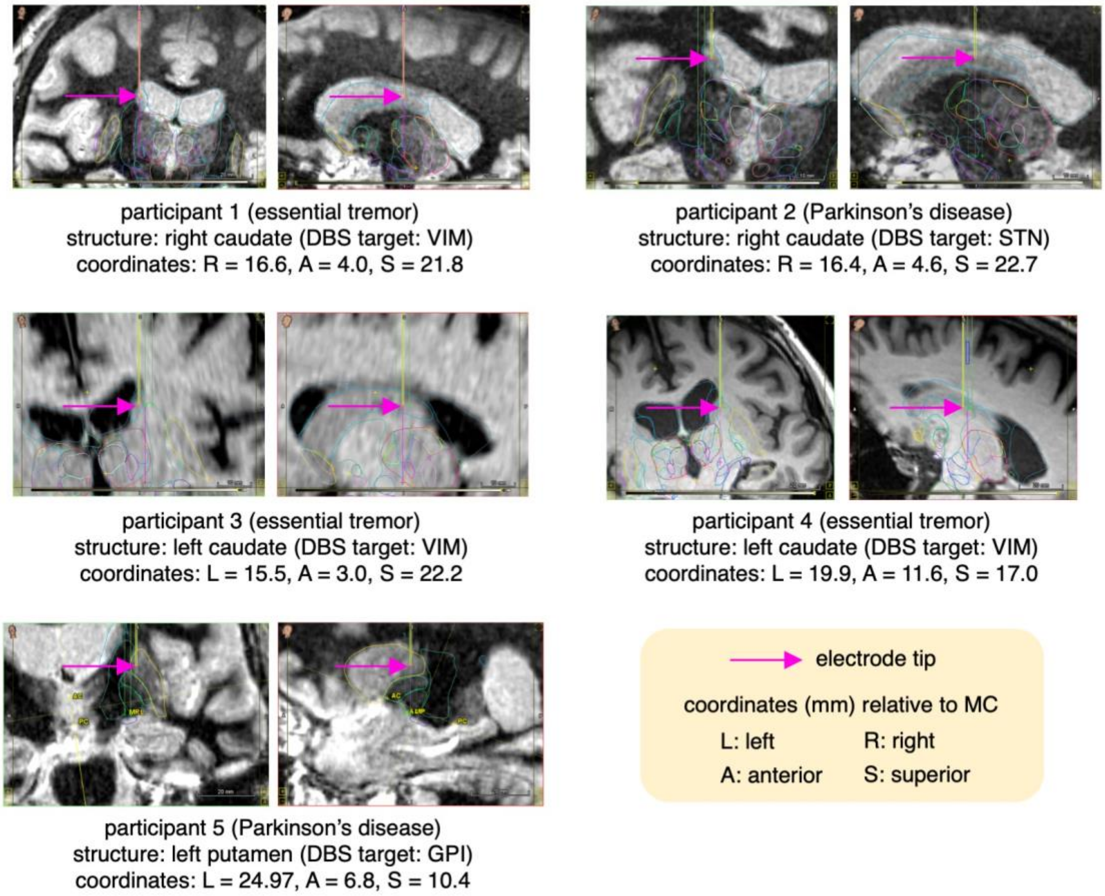

Figure S1. Electrode coordinates in participants 1-5. [Related to Figures 3-5]

Pink arrow indicates electrode tip overlaid onto structural magnetic resonance images used for surgical planning and navigation. Coordinates are relative to the mid-point between the anterior and posterior commissures (MC) – both of which are determined manually by the surgeon during surgical planning. VIM: ventral intermediate nucleus of thalamus. STN: sub-thalamic nucleus. GPI: internal segment of globus pallidus.

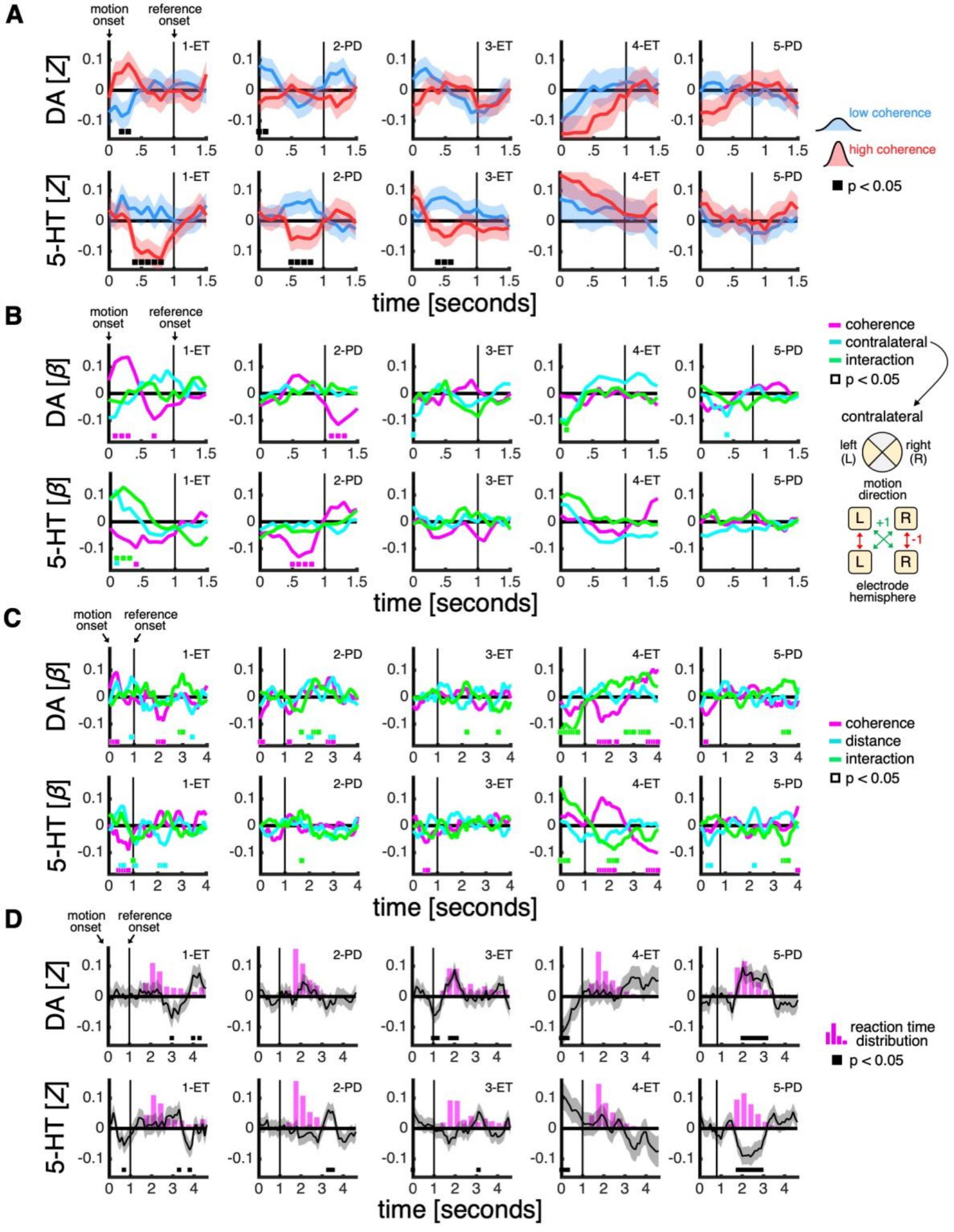

Figure S2. Dopamine and serotonin signalling in participants 1-5. [Related to Figures 3 and 5]

(A) Dopamine and serotonin signalling in relation to coherence. Marker indicates that time series for low coherence (blue) and high coherence (red) are statistically different ($p < 0.05$, independent-samples t test). Data are represented as mean ± SEM. We highlight that the neuromodulators do not separate according to coherence in participant 5 where we recorded from the putamen. The fact that we observed robust action-related responses in participant 5 indicates that the absence of an effect of coherence – consistent with a cognition-action separation between caudate nucleus and putamen – is not due to a lack of sensitivity to dopamine or serotonin.

(B) Dopamine and serotonin signalling in relation to laterality. We used a sliding-window regression approach (see **Figure 3B** for intuition) to test whether the encoding of motion coherence depended on the laterality of the motion direction relative to the hemisphere in which our electrode was located (see **Figure S1** for electrode coordinates). This analysis was restricted to trials where the motion direction was within ±45° of the horizontal meridian (approximately 50% of trials) and therefore could be classified as leftwards (L) or rightwards (R). We included coherence (pink), a variable indicating whether the motion direction was contralateral (+1) or ipsilateral (-1) to the electrode hemisphere (cyan) and their interaction (green) as predictors of interest. We also included choice accuracy and choice reaction time as nuisance variables (not shown). All predictors were *Z* scored. There were no consistent lateralised responses (cyan or green) across participants. Marker indicates that a coefficient is statistically different from zero ($p < 0.05$) as estimated by the sliding-window regression approach.

(C) Dopamine and serotonin in relation to task variables. Marker indicates that a coefficient is statistically different from zero ($p < 0.05$) as estimated by the regression approach described in **Figure 3B**. We highlight that this analysis indicates that dopamine and serotonin carry information about the task variables at the start of a trial in participant 4 (green trace).

(D) Dopamine and serotonin signalling in relation to choice submission. Dopamine and serotonin time series from caudate nucleus (1-4) and putamen (5) locked to the onset of the motion stimulus and overlaid onto distribution over choice reaction times (pink histogram). Marker indicates that a time point is statistically different from zero ($p < 0.05$, one-sample t test).

In all panels, time series were locked to the onset of the motion stimulus, spanning a period from 1 s before stimulus onset to 5 s after stimulus onset, *Z* scored separately for each trial, and smoothed using a running average (.5 s). Top right-hand corner indicates participant number and disease state (PD: Parkinson's disease; ET: essential tremor). DA: dopamine. 5-HT: serotonin.

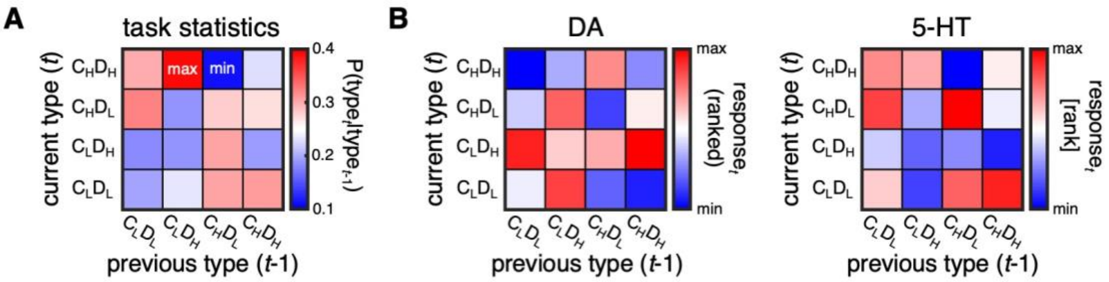

Figure S3. Experienced task statistics in participant 4. [Related to Figure 4]

(A) Experienced trial type transition probabilities. Colours denote the probability of a trial type on trial *t* (y-axis) conditional on a trial type on trial *t*-1 (x-axis). Trial type transition probabilities, $P(\text{type}_t|\text{type}_{t-1})$, were computed as the normalised counts of the number of times each trial type succeeded a particular trial type. The expected value of $P(\text{type}_t|\text{type}_{t-1})$ under randomisation is 0.25 (white).

(B) Neuromodulatory responses to trial type transitions. Colour denotes the average neuromodulatory response to a particular trial type transition. To obtain these values, we first averaged time points across a window from 0 s to 1.5 s (the period during which a trial type is revealed) within each trial and then averaged across trials within each trial type transition. Values are rank transformed for visualisation. Time series underlying this analysis were locked to the onset of the motion stimulus, spanning a period from 1 s before stimulus onset to 5 s after stimulus onset, and *Z* scored separately for each trial.

C: coherence. D: distance. L: low. H: high. DA: dopamine. 5-HT: serotonin.

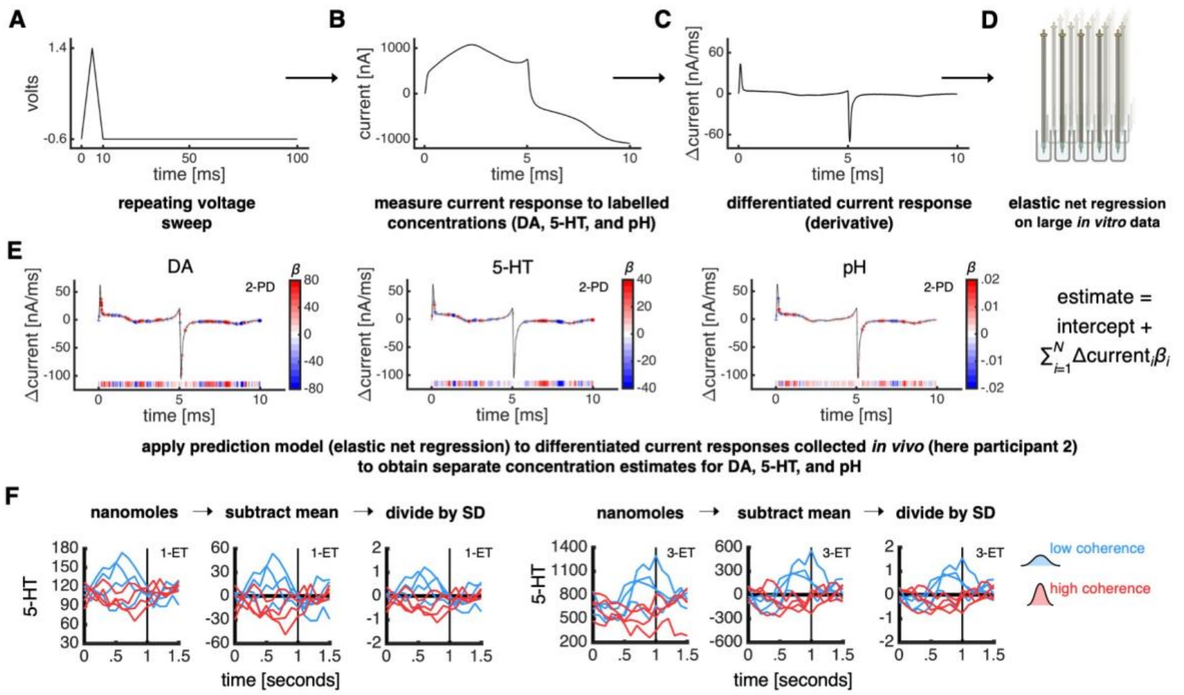

Figure S4. Illustration of dopamine-serotonin modelling. [Related to STAR Methods]

(A) Triangular voltage waveform.

(B) Example current response during the triangular voltage waveform portion of the 100 ms duty cycle.

(C) Example differentiated current response (differentiated current responses are used for both model training and concentration estimation).

(D) The prediction model is trained using cross-validated elastic net regression on large concentration-labelled data collected *in vitro*.

(E) Example differentiated current response from participant 2 with the parameter weights of their prediction model overlaid (see colour bar). We highlight that the information used to separate and estimate dopamine, serotonin, and pH is distributed throughout the differentiated current response and is not localised at singular oxidization or reduction peaks.

(F) Illustration of normalisation procedure (*Z* score) as applied to an example set of serotonin time series from caudate nucleus grouped by level of coherence. For each trial, we first subtract the mean response – to detect transient changes and facilitate comparison across trials – and then divide by the standard deviation across time points – to facilitate comparison across participants. Potential sources of differences in the mean trial response within a participant include tonic dopamine and serotonin tracking physiological states that are not relevant to our task and slow drifts in the current response of the electrode. Potential sources of differences in the mean trial response across participants include baseline dopamine and serotonin levels and the physical distance between the electrode and the sites of neuromodulator release. Notably, the normalisation procedure does not affect the shape of the neuromodulator time series but brings them into a common frame of reference for data analysis. Time series were locked to the onset of the motion stimulus (0 s), spanning a period from 1 s before stimulus onset to 5 s after stimulus onset, and smoothed using a running average (.5 s). Vertical line indicates the onset of the reference direction. Top right-hand corner indicates participant number and disease state (PD: Parkinson's disease; ET: essential tremor). 5-HT: serotonin.

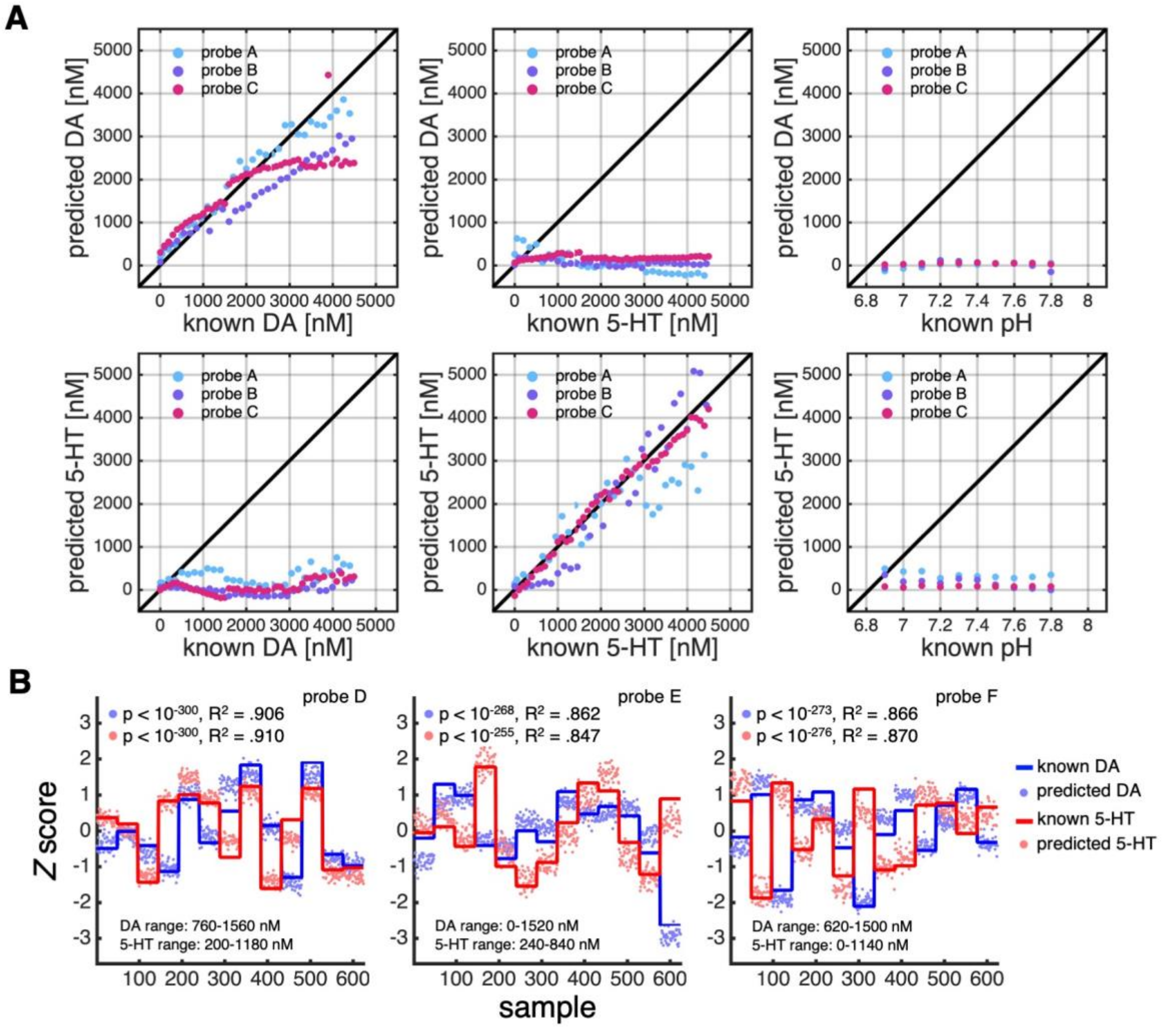

Figure S5. Evaluation of dopamine-serotonin prediction model in out-of-training *in vitro* data from six naïve probes. [Related to STAR Methods]

(A) The first three probes (A, B, C) contributed datasets that were collected in the same way as those used for training of the prediction model. We divided the DA and 5-HT datasets from the probes into three parts – each spanning a range of 1500 nM – as a given *in vivo* dataset is unlikely to span the full 0-4500 nM range. We then created predictions for each dataset and evaluated model performance by plotting predicted versus known concentrations. Each dot shows the average predicted concentration of an analyte (y-axis) as a function of the known concentration of an analyte in a given dataset (x-axis) – with each probe indicated by a unique colour. Here, we highlight two results (using [column,row] to denote plots). First, within-analyte plots (i.e., [1,1] and [2,2]) show that model predictions scale linearly with known concentrations and are close to the identity line for biologically realistic ranges (< 3000 nM). We note, however, that the exact scaling does not impact any conclusions in the current study. Our claims are based on relative changes in dopamine or serotonin at short timescales around events of interest. Second, comparison of within-analyte plots to between-analyte plots (i.e., [1,1] and [2,2] versus [1,2], [1,3], [2,1] and [2,3]) shows that the model does not confuse analytes.

(B) The remaining three probes (D, E, F) contributed datasets that were collected in solutions with a mixture of dopamine and serotonin against a stable background of pH (7.4) – with each probe exposed to a unique range of dopamine and serotonin. Each sample is a voltammogram – solid lines indicate known concentration (constant across multiple samples) and dots indicate predicted concentration. The data are displayed in units of *Z* score as our approach is optimised for detecting relative changes in concentration and in order to facilitate comparison between dopamine and serotonin (concentrations were sampled from different ranges). The relationship between known and predicted concentrations was quantified within a regression framework.

DA: dopamine. 5-HT: serotonin.

data	outcome	predictor	df.	estimate (95% CI)	t statistic	p-value
combined	accuracy	coherence	2727	0.63 (.37, .89)	4.76	< .001
		distance	2727	0.53 (.23, .83)	3.46	.001
		interaction	2727	0.25 (.06, .43)	2.65	.008
	RT	coherence	2727	-0.17 (-.27, -.08)	-3.58	< .001
		distance	2727	-0.19 (-30, -.09)	-3.57	< .001
		interaction	2727	-0.07 (-.18, .04)	-1.30	.195
	confidence	coherence	908	0.18 (.03, .34)	2.30	.022
		distance	908	0.08 (.02, .15)	2.44	.015
		interaction	908	0.04 (-.06, .13)	0.81	.418
presurgery	accuracy	coherence	1409	0.55 (.24, .85)	3.54	< .001
		distance	1409	0.46 (.19, .73)	3.32	.001
		interaction	1409	0.25 (.03, .48)	2.23	.026
	RT	coherence	1409	-0.16 (-.28, -.04)	-2.60	.009
		distance	1409	-0.15 (-31, .00)	-1.95	.051
		interaction	1409	-0.07 (-.20, .06)	-1.06	.290
	confidence	coherence	485	0.19 (.02, .36)	2.21	.028
		distance	485	0.04 (-.08, .15)	0.61	.540
		interaction	485	0.07 (-.04, .17)	1.18	.239
surgery	accuracy	coherence	1314	0.77 (.47,1.07)	4.99	< .001
		distance	1314	0.64 (.31, .97)	3.82	< .001
		interaction	1314	0.28 (.02, .53)	2.13	.034
	RT	coherence	1314	-0.19 (-.27, -.12)	-5.10	< .001
		distance	1314	-0.24 (-32, -.16)	-5.97	< .001
		interaction	1314	-0.07 (-.18, .03)	-1.32	.187
	confidence	coherence	419	0.18 (.02, .34)	2.22	.027
		distance	419	0.13 (.04, .23)	2.76	.006
		interaction	419	0.01 (-.09, .12)	0.20	.843

Table S1. Hierarchical mixed-effects regression. [Related to Figure 2]

We analysed behavioural responses using hierarchical mixed-effects regression, including participant-level intercepts and slopes. Results are shown for data combined across the presurgery and surgery sessions (yellow) and separately for each session (presurgery: green; surgery: pink).

participant	1	2	3	4	5
main figure	2 and 3	2 and 3	2 and 3	2 and 4	2 and 5
disease state	ET	PD	ET	ET	PD
recording site	caudate nucleus	caudate nucleus	caudate nucleus	caudate nucleus	putamen
medications during surgery	vancomycin<br>hydralazine<br>diltiazem<br>fentanyl<br>midazolam<br>ondansetron<br>phenylephrine	vancomycin	vancomycin<br>hydralazine	vancomycin<br>hydralazine	vancomycin
psychoactive medications withheld		citalopram<br>gabapentin<br>tamsulosin	primidone<br>trazodone		tamsulosin

Table S2. Medication status for each participant. [Related to Figures 2-5]

The table shows medications administered and psychoactive medications withheld during surgery. PD: Parkinson’s disease. ET: essential tremor.

r	$j \rightarrow$				
i<br>$\downarrow$	DA: [0, 900]<br>5-HT: [0, 900]<br>pH: [6.9, 7.8]	DA: [0, 900]<br>5-HT: (900, 1800]<br>pH: [6.9, 7.8]	DA: [0, 900]<br>5-HT: (1800, 2700]<br>pH: [6.9, 7.8]	DA: [0, 900]<br>5-HT: (2700, 3600]<br>pH: [6.9, 7.8]	DA: [0, 900]<br>5-HT: (3600, 4500]<br>pH: [6.9, 7.8]
	DA: (900, 1800]<br>5-HT: [0, 900]<br>pH: [6.9, 7.8]	DA: (900, 1800]<br>5-HT: (900, 1800]<br>pH: [6.9, 7.8]	DA: (900, 1800]<br>5-HT: (1800, 2700]<br>pH: [6.9, 7.8]	DA: (900, 1800]<br>5-HT: (2700, 3600]<br>pH: [6.9, 7.8]	DA: (900, 1800]<br>5-HT: (3600, 4500]<br>pH: [6.9, 7.8]
	DA: (1800, 2700]<br>5-HT: [0, 900]<br>pH: [6.9, 7.8]	DA: (1800, 2700]<br>5-HT: (900, 1800]<br>pH: [6.9, 7.8]	DA: (1800, 2700]<br>5-HT: (1800, 2700]<br>pH: [6.9, 7.8]	DA: (1800, 2700]<br>5-HT: (2700, 3600]<br>pH: [6.9, 7.8]	DA: (1800, 2700]<br>5-HT: (3600, 4500]<br>pH: [6.9, 7.8]
	DA: (2700, 3600]<br>5-HT: [0, 900]<br>pH: [6.9, 7.8]	DA: (2700, 3600]<br>5-HT: (900, 1800]<br>pH: [6.9, 7.8]	DA: (2700, 3600]<br>5-HT: (1800, 2700]<br>pH: [6.9, 7.8]	DA: (2700, 3600]<br>5-HT: (2700, 3600]<br>pH: [6.9, 7.8]	DA: (2700, 3600]<br>5-HT: (3600, 4500]<br>pH: [6.9, 7.8]
	DA: (3600, 4500]<br>5-HT: [0, 900]<br>pH: [6.9, 7.8]	DA: (3600, 4500]<br>5-HT: (900, 1800]<br>pH: [6.9, 7.8]	DA: (3600, 4500]<br>5-HT: (1800, 2700]<br>pH: [6.9, 7.8]	DA: (3600, 4500]<br>5-HT: (2700, 3600]<br>pH: [6.9, 7.8]	DA: (3600, 4500]<br>5-HT: (3600, 4500]<br>pH: [6.9, 7.8]

Table S3. Grid of concentration ranges used to train dopamine-serotonin prediction model. [Related to STAR Methods]

DA: dopamine. 5-HT: serotonin.

neuromodulator	participant	percentile [nM]						
		1st	5th	25th	50th	75th	95th	99th
DA	1 (ET)	-80	-56	-23	0	23	56	79
	2 (PD)	-644	-357	-191	0	188	458	644
	3 (ET)	-188	-143	-62	0	60	142	193
	4 (ET)	-328	-170	-64	-1	63	166	262
	5 (PD)	-170	-121	-49	0	49	122	176
5-HT	1 (ET)	-88	-62	-25	0	26	62	87
	2 (PD)	-196	-137	-56	0	56	137	196
	3 (ET)	-858	-607	-246	1	243	612	895
	4 (ET)	-964	-488	-125	13	150	451	973
	5 (PD)	-203	-142	-57	1	57	140	199

Table S4. Range of changes in dopamine and serotonin levels within a trial for each participant. [Related to STAR Methods]

The table shows percentiles for changes in neuromodulator levels within trials relative to average trial responses (calculated for stimulus-locked time series). PD: Parkinson’s disease; ET: essential tremor. DA: dopamine. 5-HT: serotonin.
